# Genome Sequences of *Streptomyces* Cluster BE2 phages CeilingFan and Mugiwara

**DOI:** 10.17912/micropub.biology.001971

**Published:** 2026-05-06

**Authors:** Imade Y. Nsa, Ezichukwu E. Ejiofor, Emmanuel T. Ajayi, Ifunanya O. Okoli, Chibueze S. Uchegbu, Opeoluwa S. Aworetan, Fuad T. Jebe-Abdullah, Hauwa J. Kamselem, Shina O. Abidoye, Evans C. Oleka, Oluwatunkunmi J. Okupa, Fuhad T. Lawal, Grace O. Immanuel, Tomi E. Adeyanju, Faith O. Onyekachi, Omogwigho G. Akpederi, Oluwafoyinsolami I. Adisa, Modesola O. Onikoro, Chase Boyles, Julie A. Merkle, Matthew O. Ilori

**Affiliations:** 1 Department of Microbiology, University of Lagos, Lagos, Nigeria; 2 Department of Biology, University of Evansville, Evansville, Indiana, United States

## Abstract

CeilingFan and Mugiwara are novel BE2 cluster siphoviruses isolated from soil samples on *Streptomyces griseus* ATCC 10137 and *Streptomyces avermitilis* NRRL 8165, respectively. CeilingFan has a genome of 132,946 bp with 49.3% G+C content, and Mugiwara has a genome of 133,330 bp with 49.1% G+C content. Both phage genomes encode 44 tRNAs and one tmRNA. The phages are assigned to the BE2 cluster of actinobacteriophages based on gene content similarity.

**Figure 1. Plaques and Transmission Electron Micrographs of Mugiwara (left), and CeilingFan (right):** (A) Plaques formed by phage Mugiwara on *S. avermitilis* NRRL 8165. (B) Plaques formed by phage CeilingFan on *S. griseus* ATCC 10137. (C) Transmission electron micrograph of Mugiwara stained with 1% uranyl acetate and imaged at 120 kV, Scale bar = 75 nm. The capsid measures 79 nm, and its tail length is 348 nm, (n=1). (D) Transmission electron micrograph (TEM) of CeilingFan stained with 1% uranyl acetate using JEOL 1400 Plus and imaged with AMT8 mixpel camera at 100 kV, Scale bar = 100 nm; the capsid diameter is [62.2 nm + 4.76, (n=10)], and the tail length is [365nm + 15.6, (n=10)].

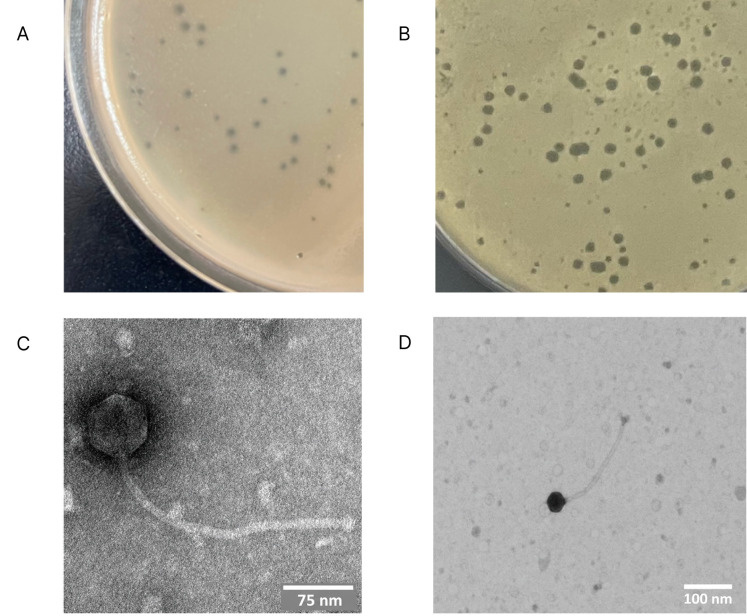



## Description

The continued discovery and characterization of *Streptomyces* bacteriophages improve our understanding of phage-host interactions, phage diversity, phage genome architecture, and their potential applications in combating antibiotic resistance. *Streptomyces griseus* subsp. *griseus* ATCC 10137 and *Streptomyces avermitilis* NRRL 8165 are bacterial type strains for studying the biology of *Streptomyces* (Hatfull, 2020; Kronheim et al., 2023; Rackow et al., 2024).

Here, we report the characteristics of CeilingFan and Mugiwara, isolated from slightly damp soil in Evansville, IN, USA (37.973088 N, 87.533631 W) and a grassy area near fir trees in Noertrange, Luxembourg (49.98127, 5.903158 E), respectively. Using standard protocols (Zorawik et al., 2024), CeilingFan was extracted by the enrichment method and Mugiwara by direct isolation. Briefly, soil samples were suspended in nutrient broth supplemented with glucose, MgCl2, and Ca (NO3)2, and the suspension was subsequently filtered using a 0.22 µm filter. The filtrate for Mugiwara was plated directly in top agar supplemented with *S. avermitilis* NRRL 8165, whereas the filtrate for CeilingFan was first inoculated with *S. griseus* ATCC 10137, incubated at 20 °C for 72h, and the resulting culture filtered before the filtrate was plated with *S. griseus* ATCC 10137. Plates for Mugiwara and CeilingFan were always incubated at 37 ˚C and 30 ˚C, with CeilingFan plates incubated for 48 hours. Mugiwara formed small, clear plaques (1.14 mm + 0.63, n=41 (Schnieder et al., 2012)), whereas CeilingFan formed slightly larger, clear plaques (1.9 mm + 0.63, n=64 (Cacciabue et al., 2019)). Both phages were purified through three rounds of plating before lysates were prepared. Transmission electron microscopy after negative staining of lysates with uranyl acetate (1%) revealed siphovirus morphology for both phages. CeilingFan has a capsid diameter of 62 nm and a tail length of 346 nm (n=10), while Mugiwara featured a larger capsid measuring 79 nm and a tail length of 348 nm (n=1) (Figure 1).

DNA was extracted from high-titer lysates of CeilingFan and Mugiwara using the Promega Wizard DNA Cleanup kit. CeilingFan and Mugiwara genomes were prepared for sequencing using the NEB Ultra II Library kit (New England Biolabs, Ipswich, MA, USA) and sequenced on the Illumina MiSeq 1000 v3 platform using the 150-bp single-end sequencing strategy at the Pittsburgh Bacteriophage Institute. Raw reads were assembled using Newbler version v3.0 (Miller et al., 2010) and checked for completeness and genomic termini using Consed v29 (Gordon et al., 1998; Russell, 2018). The number of reads generated for CeilingFan was 413,717, allowing for assembly with 466-fold coverage of a genome 132,946 bp long, with G+C content of 49.3% and a direct terminal repeat length of 12,339 bp. For Mugiwara, 342,705 reads provided 391-fold coverage of an assembled genome 133,330 bp long with G+C content of 49.1% and a direct terminal repeat length of 11,945 bp.

Auto-annotation was performed in DNA Master v 5.23.6 (http://cobamide2.bio.pitt.edu/) with Glimmer v3.02 (Delcher et al., 2007) and Genemark v2.5 (Besemer and Borodovsky, 2005), then manually refined using Starterator (http://phages.wustl.edu/starterator) for start sites comparisons (Pope and Jacobs-Sera, 2018). Functions were predicted by BLAST, using the Actinobacteriophage and NCBI non-redundant database searches v2.13.0 (Altschul et al., 1997; Russell and Hatfull, 2017), HHPRED, using the PDB_mmCIF70, (Söding, 2005), Pfam- v.36, NCBI Conserved Domains databases, Phamerator, using Actino_draft database v578 (Cresawn et al., 2011), The Observable Notebook, Subclusters (Bendele et al., 2025) and The Observable Notebook, Pham Matrices (Cobb et al., 2025). The presence of tRNAs and tmRNAs were examined using Aragorn v1.2.41 (Laslett and Canbett, 2004) and tRNAscan-SE v2.0.12 (Lowe and Eddy, 1999) and membrane proteins were identified using SOSUI v1.1 (Hirokawa et al., 1998; Mitaku & Hirokawa, 1999; Mitaku et al., 2002) and DeepTMHMM 1.0.42 (Hallgren et al., 2022). All software was used with default parameters.

Based on gene content similarity (GCS) of at least 35% to previously annotated phages within the Actinobacteriophage Database (phagesdb.org), both phages were assigned to subcluster BE2 (Russell and Hatfull, 2017; Pope et. al., 2017) and share 79% GCS with one another. CeilingFan encodes 44 predicted tRNAs, one predicted tmRNA and 248 predicted protein-coding genes, with 64 of the latter assigned putative functions; 181 genes are in the forward direction and 67 genes in the reverse direction. Mugiwara similarly encodes 44 putative tRNAs and one tmRNA and has 247 predicted protein-coding genes, of which 59 could be assigned putative functions; 183 genes are in the forward direction and 64 reverse genes.

As is typical for BE2 phages, genes encoding putative holin and endolysin proteins were identified in both CeilingFan and Mugiwara. No genes encoding a putative integrase or immunity repressor were identified in either phage, suggesting they are unlikely to establish lysogeny. MazG-like nucleotide, Ro-like RNA binding protein, DprA-like ssDNA binding protein, and Cas4 exonuclease were among the functions commonly identified across BE2 phages. Mugiwara lacks the Lys-M-like peptidoglycan binding protein found in a majority of BE2 phages (23/33 phages) but it is the only BE2 member in which a putative histidine triad nucleotide binding protein has been identified. Uniquely, Mugiwara encodes a putative thioredoxin that shares little homology to the thioredoxin encoded by other BE2 phages. Similarly, CeilingFan encodes an HNH endonuclease and DNA Q-like DNA polymerase III subunit with little homology to that encoded by other BE2 phages, except for phage TomSawyer.

**Nucleotide sequence accession numbers**

The complete genome sequences for both phages are available at GenBank with Accession Nos. PV165877 and PV876970 and Sequence Read Archive (SRA) Nos.SRR34858420 and SRR34858425 (CeilingFan and Mugiwara respectively).
